# Reproductive Health Services Utilization and Associated Factors Among Adolescents in Anchar District, East Ethiopia 

**Published:** 2017-06

**Authors:** Mustafa Geleto Ansha, Challi Jira Bosho, Fikru Tafese Jaleta

**Affiliations:** 1 Department of Public health, Debre Berhan University, Debre Berhan, Ethiopia; 2 Department of Health Services Management, Jimma University, Jimma, Ethiopia

**Keywords:** Adolescents, VCT, Reproductive Health, Population Fund (FP), Sexually Transmitted Infection (STI), Utilization

## Abstract

**Objective:** To assess reproductive health service utilization and associated factors among adolescents in Anchar District, West Hararghe Zone, Oromia Region, East Ethiopia.

**Materials and methods:** A community based cross-sectional study using quantitative and qualitative method of data collection was applied from March 1to 30, 2013. Simple random sampling method was used for quantitative and Purposive sampling technique used for qualitative method. Four hundred two adolescents were interviewed for quantitative study. Four focus groups and ten in-depth interviews were conducted for qualitative study. Binary and Multiple logistic regressions were used for association at p < 0.05 using SPSS Version 16.0 software. Qualitative data was transcribed, and result was presented by narration.

**Results:** Forty two (39.3%) female adolescents have ever used family planning. One hundred eight four (45.8%) adolescents have ever used VCT services. Males were 5.25 times more likely to use VCT than females (AOR = 5.25,C.I = 1.07, 25.87) and those perceived themselves as high risk for HIV were 8.22 times more likely to use VCT than their counterparts (AOR = 8.22, C.I = 1.065, 35.49). Lack of adolescent reproductive health services, Harmful Traditional Practices, lack of privacy and inconvenient service hour were reasons for not utilizing the service.

**Conclusion:** More than half of adolescents were not utilizing family planning, and VCT services. Therefore, intensified effort is needed to increase utilization of these services for adolescents.

## Introduction

World Health Organization defines adolescents as persons with the age group of 10-19 years (1). Adolescence is the period of transition from childhood to adulthood which is characterized by spurts of physical, mental, emotional and social development (2) with experimentation and engagement of a wide range of behaviors (3).

Recently adolescents number is estimated to be 1.25 billion globally (4) of which, 513 million are 15-19 years old (5) and 85% of them in developing countries (6). They are most vulnerable to a range of reproductive health problems, such as too-early pregnancy and childbearing; unsafe abortion and sexually transmitted infections (STIs) including HIV (7).

According to the World Health Organization about one half of all HIV infections worldwide occur among people aged less than 25 years and up to 100 million adolescents become infected with a curable sexually transmitted diseases, best estimates indicate that about one out of twenty adolescents in the world contract sexually transmitted diseases each year (5, 6, 8). Adolescents’ use of contraceptive method is generally low and they are less likely to use condoms than adults because of lack of access and, for girls in particular, the inability to insist on their use and make informed decisions and choices (9, 10).

In developing countries there are about 12.8 million births among adolescents aged 15-19 years and a large proportion of these pregnancies are unplanned (11). Each year more than 2 million adolescents undergo unsafe abortion and for a variety of reasons adolescents are more likely to seek abortions later in their pregnancies than adult women (7, 8, 10). Studies in sub-Saharan Africa revealed that adolescent women accounted for as many as 60 percent of those hospitalized for abortion complications (10).

Each day 500,000 young people are infected with STI and in Africa the WHO estimates that 60% of all new HIV infections occur in adolescents aged 15-19 years (12). Studies in Nigeria and India showed that majority of adolescents those who contracted STI or other reproductive health problems did not use reproductive health services for several reasons including shame and embarrassment, high cost of service, negative provider attitudes and perceived lack of Confidentiality, some feared being ridiculed and discrimination they face by some health service staff in accessing contraceptive, sexually transmitted infection services or other services (13).

Many young girls of Ethiopia engage in sex at a relatively early age, and they are unlikely to use contraception (14) as a result becomes vulnerable to unintended pregnancies which is more than one in three births to women aged 15-19 years. Forty five percent of the total births in Ethiopia occur among adolescent girls and young women. The number of births among women aged 15- 19 years is also 99 per 1,000 women (15, 16). Contraceptive use among currently married women show that only 23% of aged 15-19 years use modern contraceptive which is lower compared with other age groups. Sexually active youth aged 15-19 years those who have been tested for HIV in the past 12 months were only 23.9% and 26.9% women and men respectively (16). In addition self reported prevalence of STI symptoms among sexually active adolescents aged 15-19 years show that 1.4% and 0.5% women and men respectively have experienced STI symptoms (17).

## Materials and methods

The study was conducted in Oromia regional state Anchar district, west Hararghe zone, east Ethiopia, 300 KM from Addis Ababa. According to the 2007 Ethiopian Housing and Population census, the district has an estimated total population of 96,382 in 2013. There are five health centers, twenty three health posts and five private clinics; and the potential health services coverage of the district is more than 100% in 2013 year.

A community based cross-sectional study was conducted from March 1 to 30, 2013 using both qualitative and quantitative data collection methods. The sample size for quantitative was determined by using a single population proportion formula considering proportion of family planning service utilization 50%. Initially lists of adolescents obtained from woreda administration and serial number was given for each adolescent to get sampling frame. Then the serial numbers entered into SPSS version 16.0 and generate random number; adolescents correspond to generated serial number were contacted for face to face interview. For qualitative four focus groups were conducted. Additionally 10 individuals were selected for in-depth interview. Both of them selected purposefully assuming theotical saturation of idea. Quantitative data was collected by using a structured and pre-tested interviewer administered questionnaire. Semi-structured open ended interview and FGD guide were designed for qualitative data collection. Data were checked for completeness, cleaned, coded and entered into epidata version 3.1, and then exported to SPSS version 16.0 for further analysis. Frequencies were used to summarize descriptive statistics. Binary and multiple logistic regression analysis were used to assess variables that have association with utilization of reproductive health services among adolescents. For qualitative findings data was first transcribed, edited, compiled manually and the result was presented by narration. Ethical clearance was obtained from the Ethical review committee of College of Public Health and Medical Sciences of Jimma University after assessing the study against Ethical standard under CPHMS/0443/2013 code, verbal consent was obtained from each study participants and their parents.

## Results

Four hundred two adolescents aged 15-19 years were participated in the study. 

**Table 1:** Female adolescents’ lifetime and current utilization of family planning, Anchar district, West Hararghe Zone, Oromia region, East-Ethiopia, March 2013 (n = 107)

	**Frequency**	**(%)**
Ever used family planning		
Yes	42	39.3
No	65	70.7
Contraceptive use during their first sexual		
Intercourse		
Yes	34	31.8
No	73	68.2
Contraceptive use during their last sexual		
Intercourse		
Yes	22	20.6
No	85	79.4
Reason for not utilizing the service (N = 76)		
Too young	4	5.3
Feel afraid/embarrassed to get service	4	5.3
Lack of knowledge how to use contraception	18	23.7
Method		
Religious opposition	21	27.6
Opposition from sexual partner	9	11.8
Fear of side effects	2	2.6
Lack of preferred methods	6	7.9
Lack of privacy by health workers	12	15.8

Two hundred twenty one (55%) of them were male, 360 (82.1%) were never married, and 246 (61.2%) were Muslims. Most of respondents (81.8%) are currently in the school, 248 (61.7%) live with both parents, 335(83.3%) and 237(59%) adolescent mother and father could not read and write. The median age of mothers and fathers was 40 and 45 years respectively.

Two hundred thirty six (58.7%) of them had sexual experience and 225 (56.0%) adolescents were sexually active in the past twelve months. Three hundred forty two (85.1%) reported that they were highly followed by their parents.

From the total female adolescents who had sexual intercourse in their lifetime 42(39.3%) have ever used family planning services, among those who have not utilized family planning services; 21(27.6%) religious opposition 18 (23.7%) lack of knowledge how to use contraception method, and 12 (15.8%) lack of privacy were reported as a reasons (Table 1).

Fifteen respondents have an experience of at least one of the STI symptoms; of them ten have sought STI treatment services while the rest have not attended any STI treatment services. The reason for not seeking the service was feeling of afraid or being embarrassed to get the service (Table 2).

Eighty two (31.5%) respondents perceived that they are at high risk of contracting HIV. One hundred eighty four (45.8%) of them have ever used VCT service (Table 3). One hundred thirty one (60.1%) responded feel of afraid/embarrassed and twenty five (10.5%) responded lack of confidentiality by health workers as reason for not utilizing (Table 3).

Religion and ever discussion about family planning services have significant association with ever use of family planning. Orthodox Christian followers were 2.45 times more likely to ever use family planning compared to Muslims (AOR = 3.45, C.I = 1.23, 9.68) and adolescents those ever discussed about family planning were 12.98 times more likely to ever use family planning compared to their counterparts (AOR = 12.98, C.I = 4.63, 36.40) (Table 4).

Sex and magnitude of perception of risk for HIV were independently associated with ever use of VCT service after controlling effect of other covariates.

Males were 5.25 times more likely to ever use VCT service compared to females (AOR = 5.25, C.I = 1.065, 25.87) and adolescents who were perceived themselves as high risk were 8.22 times more likely to ever use VCT compared to those perceived themselves as low risk for HIV/AIDS (AOR = 8.22, C.I = 1.07, 35.74) (Table 5).

**Table 2:** Respondents’ characteristics regarding to awareness and utilization of STI case management services Anchar district, west Hararghe zone, Oromia region, East-Ethiopia, March, 2013 (N = 402)

	**Frequency**		**(%)**
Ever heard about STI case management service			
Yes	336		83.6
No		66	16.4
Awareness about place where STI case management services available			
Yes		321	79.9
No		81	20.1
Ever Discussed about STI case Management			
Yes		247	61.4
No		155	38.6
Source of information(N = 336)			
Radio		231	68.8
Television		26	7.7
News paper		8	2.4
Peer groups/friends		6	1.8
Teacher		21	6.2
Health workers		44	13.1
Have experience of the following symptoms			
Burning sensation during urination		7	1.7
Bad smelling genital discharge		4	1.0
Genital ulcer/sore		3	0.7
Itching around genitalia		1	0.2
Did not experience any of above symptoms		387	96.3
If have the above symptoms have seek the service			
Yes		10	66.7
No		5	33.3
Reason for not seeking the service			
Feel of afraid/embarrassed		5	100

The key informants explained many factors for under utilization of reproductive health services by adolescents; these are: fear of social value and being embarrassed, misconception of adolescents about pregnancy, unsafe sex, shortage of supply, harmful traditional practices and lack of school based adolescent reproductive health services were among reason explained by both health workers and school teachers.

The expert from health center said: *“…when we ask adolescents who come for abortion some of them explain that they have conducted only one sexual intercourse and they have perceived that they can’t become pregnant in a single intercourse… others said they have made sexual intercourse without condom because their partners refused to use it…, others explained that have engaged in the sexual activity unintentionally…and there is also fear of embarrassment...” (Nurse, 31 years old)*

All experts also raised shortage of some contraceptives such as Depo-Provera in the last year. The summary of information is: *“Last year there is inconsistence supply of family planning drugs... especially Depo-Provera was not available for more than three months consequently…and sometimes the syringe will not available with drug…”*

Lack of school based reproductive health education and club was reason mentioned by teachers. The 27 years old high school teacher said that: *“there was no adolescent reproductive health clubs in the school, but most of our students are in the fire age; unless reproductive health education is given for them they are easily engage in unsafe sexual intercourse”*

All health extension workers participated in in-depth interview raised the issue of early marriage as one factor for not utilizing reproductive health.

**Table 3:** Characteristics of respondents regarding to VCT service awareness and utilization Anchar district, west Hararghe zone, Oromia region, East-Ethiopia, March, 2013

		**Frequency**	**(%)**
Ever heard about VCT service			
Yes		377	93.8
No		25	6.2
Awareness about place where VCT			
services available (N = 402)			
Yes		377	93.8
No		25	6.2
Ever Discussed about VCT (N = 402)			
Yes		300	74.6
No		102	25.4
Source of information (N = 377 )			
Radio		244	64.6
Television		12	3.2
News paper		2	0.5
Peer groups/friends		9	2.4
Teacher		11	2.9
Health workers		99	26.3
Perception of risk for HIV(N = 402)			
Yes		261	64.9
No		141	35.1
Magnitude of risk (N = 261)			
High		82	31.5
low		178	68.5
Ever utilized VCT service			
Yes		184	45.8
No		218	54.2
Where you get the service (N = 184)			
Health center		177	96.2
Private clinic		3	1.6
Others*		4	2.2
Reason For not Utilizing Visit Service			
Fear of afraid/embraced to get service		131	60.1
Lack of confidentiality by health workers	25		11.4
Inconvenient service hour		18	8.3
Long waiting time		18	8.3
Judgmental attitude by health workers		2	0.9
Others**		24	11

* In the school, at the health post;

** Too young to use service, lack of knowledge to use the service

Among them one 24 years old health extension worker said that: *“presence of deep-rooted harmful traditional practices like ‘Chebsa’* *and ‘Telefa’ type of marriage also hinders the use of reproductive health for two reasons: primarily the marriage was not based on the willingness of adolescent so that no one give chance for blood test, secondly the marriage by itself is immediate/emergency and there was no time for screening.”*

Result of FGD indicates relationship as boy/girlfriend, misconception about risks, lack of adolescent friendly services and harmful traditional practices as reason for not utilizing the services.

The summary of their idea is here: “*Working hours of the health institutions is one problem; since they work during school hours you must miss the day’s classes to get the service…when you go to health facilities you are treated with adults there is no privacy… you may meet someone whom you know…relationship as boyfriend/girlfriend without understanding reproductive health problems…adolescents forced by their partners to have sexual intercourse without condom….”*

Additionally female discussants added that: “…*some adolescents perceived that they can’t become pregnant in the single sexual intercourse… some of them even forced by their partners not to use family planning.”*

Females FGD out of school explained harmful traditional practice like ‘*Chebsa and Telefa’ *as major reason for not utilization of the services*. *The summary of discussion was as follows: *“…adolescents marry to the person who she do not know, and most of them marry by ‘Chebsa’ while others by ‘Telefa’… no one allow them to have time for blood test… marriage is not based on the willingness of adolescent.”*

**Table 4:** Factors associated with ever use of FP services among female adolescents in Anchar district, west Hararghe zone, Oromia region, East-Ethiopia, March, 2013

**Ever used FP**	**Yes (%)**	**No (%)**	**Crude OR ** **(95% C.I)**	**p-value**	**Adjusted OR ** **(95%. C.I)**	**p-value**
Religion						
Muslim	16 (38.1)	41 (63.1)	1.00		1.00	
Orthodox Christian	20 (47.6)	19 (29.2)	2.70 (1.15, 6.33)	0.023*	3.45 (1.23, 9.68)	0.02*
Other Christians	6 (14.3)	5 (7.7)	3.08 (0.82, 11.51)	0.095	4.08 (0.82, 20.32)	0.082
Parental Monitoring						
High	35 (83.3)	37 (56.9)	3.74 (1.47, 9.77)	0.006*	6.5 (0.99, 42.76)	0.05
Low	7 (16.7)	28 (43.1)	1.00			
Ever discussed about family planning						
Yes	35 (83.3)	20 (30.8)	11.25 (4.28, 29.62)	0.000**	12.98 (4.63,36.40)	0.000**
No	7 (16.7)	45 (69.2)	1.00		1.00	

* Significant association = p < 0.05,

** p < 0.005, reference category = 1.00

**Table 5:** Factors associated with ever use of VCT service among adolescent in Anchar district, west Hararghe, Oromia, Ethiopia, March, 2013

**Ever used VCT**	**Yes (%)**	**No (%)**	**Crude OR ** **(95% C.I)**	**p-value**	**Adjusted OR ** **(95%. C.I)**	**p-value**
Sex						
Male	119 (64.3)	102 (47.0)	2.03 (1.36,3.04)	0.001**	5.25 (1.065, 25.87)	0.042*
Female	66 (35.7	115 (53.0)	1.00		1.00	
Educational status						
Unable to read and write	13 (7.0)	22 (10.1)	1.00		1.00	
can read and write	6 (3.2)	3 (1.4)	3.39 (0.72, 15.89)	0.122	0.53 (0.022, 12.90)	0.695
Primary(1-8)	60 (32.4)	107 (49.3)	95 (0.45 ,2.02)	0.892	1.33 (0.034, 52.39)	0.880
Secondary and above(>9)	106 (57.3)	85 (39.2)	2.11 (1.01, 4.44)	0.049*	2.11 (0.51, 8.74)	0.301
Magnitude of perception of risk						
High	68 (47.6)	15 (12.7)	6.23 (3.31, 11.73)	0.000**	8.22 (1.07, 35.49)	0.005**
Low	75 (52.4)	103 (87.3)	1.00		1.00	

* Significant association = p < 0.05,

** p < 0.005, reference category = 1.00

Finally they have suggested availability of local information center for reproductive health and adolescent friendly services to increase its utilization. The summary of information was: *“…there should be local adolescent reproductive health program such as youth and adolescents’ reproductive health club both in the school and out of school…We need health services that was designed and organized for adolescents… The service has to be friendly and appropriate for youths and adolescents that work at convenient hours for students, which is safe, and which should keep privacy of clients.”*

## Discussion

This community based cross-sectional study tried to assess SRH utilization and range of factors associated with utilization of adolescents reproductive health services including socio- demographic, adolescents’ sexual activity and parental monitoring; and adolescents’ awareness about the services in the rural kebeles of Anchar district, East-Ethiopia. So far most studies on this subject have been limited to the urban areas of the country with little information on the rural areas like Anchar district.

Ever use of family planning was measured by asking adolescent use of at least one type of family planning method in their life time. In this study, it was learnt that among sexually experienced females forty two (39.3%) have ever used family planning. This result was higher than study conducted in East Gojjam (21%) and Jimma (17.6%) (18, 19). The reason might be the time difference between two studies; currently health extension workers are serving the rural community but the previous studies were conducted before implementation of health extension program. The second reason especially for East Gojjam is the percentage of married adolescents (44%) which affect the utilization as contraceptive use among married women in East Gojjam is only 12% compared to unmarried which is 57%; additionally the potential health coverage of Anchar district may facilitate for adolescents to access and utilize the service. The study conducted in Kenya showed almost similar finding which is 46.7% of females has ever used family planning (20). But this study is less than the study conducted in Brazil and Addis Ababa which showed that among sexually active female student adolescents living in Brazil 62.1% of them are using some kind of contraceptives, similarly among sexually active adolescents in Addis Ababa 67% males and 51% females have ever used condom (21,22). The reason for the difference may be the previous studies were conducted in the urban slums while the current study was focused on the rural adolescents. Additionally Brazil has promoted public campaigns to inform youngsters about the use of contraceptives but this was not conducted in our country especially for rural adolescents.

Globally more than 10% of all births or an estimated of 14 million adolescent girls between the ages of 15 and 19 years gave birth every year and within this age category over four million women have abortions, 40% of which are performed under unsafe conditions (7, 8, 10). So the use of family planning plays the key role in preventing unintended pregnancies; and consequently, minimizes unsafe abortion. In the present study among sexually active female adolescents only 39.3% have ever used family planning. This may facilitate the rapid increase of unsafe abortion as most of adolescents are unmarried and sexually active.

Adolescent discussion about family planning was strongly associated with family planning utilization. In the present study those ever discussed about family planning were almost thirteen times more likely to ever use family planning than their counterparts. This was also supported by study conducted in United States (23). The reason is due to the fact that those discussed about the services might insight deeper understanding of benefits of the service which may encourage them to use the service compared to their counterparts.

The current study revealed that there was a disparity in utilizing of family planning among orthodox Christian followers and Muslims. In generally Orthodox Christian religion followers were almost three and half times more likely to use family planning compared to Muslims. This association was not mentioned in the previous studies which might be unique for this study. This evidence is also supported by evidence from qualitative finding obtained from key informant’s in-depth interview which showed Muslim followers in rural setting refuses to use contraceptive.

In this study socio-demographic factor such as marital status, schooling status and educational level did not showed significant association with family planning service utilization. This is not consistence with study conducted in Uganda and Kenya (20, 24). This might be due to the fact that most adolescents in this area were in the school and unmarried as compared to Kenya which is 40.1%.

From the total adolescents fifteen of them have experienced with at least one of the STI symptoms, out of them ten have seek STI treatment services while the rest have not attended any STI treatment services. This result was not comparable to other studies due to the fact that small number of outcome variable which might exaggerate the percentage. However the reason responded by participants for not seeking the service was feeling of afraid or embarrassed to get the service.

Adolescents use of VCT service was 45.6%, and the result of the current study was higher than study conducted in North and south Nigeria (1.7% and 3.7%), Butajira (6%), Eastern Cape of South Africa(10.9%), Burkinafaso (1.5% for male and 2.5% for female), Ghana(1.2% for males and 2.8% for females), Malawi(4.85% for males and 3.5% for females), Uganda(2.2% for males and 10.7% for females) and Lagos(1%) (25-29). The reason might be the presence of VCT campaign in the last year and quarterly outreach program which may increase number of service users as explained by experts during in-depth interview with service providers. However the utilization of service by adolescents was remained still less than half, which needs more attention to improve the utilization coverage.

The frequently explained reasons for those who have not utilized the service were fear/embarrassment to get the services and lack of confidentiality. This is similar with study conducted in Nigeria (13). Beside these harmful traditional practices like ‘Chebsa and Telefa’ and absence of reproductive health education in the school were frequent reasons addressed through FGD.

Sex of adolescent is an important predictor for the utilization of VCT service in this study. In general, males were more likely to use VCT service as compared to females. This result is consistent with previous study done in North and South Nigeria (25).

Adolescent perception about risk of HIV has positive association with VCT service utilization. This means adolescents who perceived themselves at high risk for HIV were more likely to utilize VCT service compared to their counterparts. This result was consistent with study conducted in North and South Nigeria (25). This might be adolescent who perceived that they are at the risk of HIV check their status regarding to exposure of the HIV so that it probably increased utilization of the service when compared to those perceived as low risk.

Teachers play the negligent role in information dissemination; even though school based adolescent health service is very important for improvement of adolescent reproductive health. Lack of reproductive health club is also reason explained by teachers through qualitative response, so that the role and involvement of teachers in school based health education is less. Relationship as boy friend/girl friend without using VCT service was reason explained by school FGD but ‘Chebsa’ and ‘Telefa’ was reason explained by out of school FGD for not utilizing the service. The limitation of this study was liability to social desirability bias even though sex and age matched data collectors were used, additionally information about STI cases was based on self reported symptoms which may not pick the exact case of STI. As strength the study used mixed data collection method to strengthen the reliability of evidence.

In conclusion utilization of reproductive health services regarding to family planning and VCT by adolescents is low in general, as clearly depicted that more than half adolescents were not utilizing the services. Lack of adolescent reproductive health club, harmful traditional practices like chebsa and telefa, shortage of supply, fear/embarrassment and convenient service hour were reasons for not utilizing the service.

***Conclusion:*** Thus, intensified effort was needed to improve utilization of reproductive health among adolescents in rural setting. There should be health promotion activities in order to reach every segment of adolescent population. There should be adolescent friendly services for rural adolescent. Establishing and strengthening adolescent reproductive health club in and out of school play great role in changing behavior and increasing utilization of reproductive health.

## References

[1] Erosen J (2004). Adolescent Health and Development 'A Resource Guide for World Bank Operations Staff and Government Counterparts.

[2] WHO (1986). Young people’s health – a challenge for society, Report of a WHO health group on young people and health for all by the year 2000.

[3] Schwarz SW (2010). Adolescent Reproductive and Sexual Health, Facts for Policymakers.

[4] WHO (2008). Promoting Adolescent sexual and reproductive health through schools in low income countries; an information brief Department of child and adolescent health.

[5] Kirby D (1994). A Proposed adolescent reproductive health initiative.

[6] Belachew T, Nigussie S, Mengistie B (2003). Manual on Reproductive Health.

[7] Ingwersen R (2001). Adolescent reproductive and sexual health in the developing world.

[8] (1997). Family health international, Adolescent reproductive health, network.

[9] Karl L D, Gabriele R (2005). Sexually transmitted infections among adolescents the need for adequate health services.

[10] De BruynMaP (2004). Adolescents unwanted pregnancy and abortion. Policies, counseling and clinical care.

[11] Neelofur-Khan D (2007). Adolescent pregnancy: unmet needs and undone deeds: a review of the literature and programmes.

[12] Nwobodo E (2002). Reproductive health issues of the adolescent in Africa: Review and commentary. Nigerian Journal of Physiological Sciences.

[13] Kate Wood PA Promoting Young People’s Sexual and Reproductive Health: Stigma, Discrimination and Human Rights.

[14] Govindasamy P, Kidanu A, Bantayerga H (2002). Youth Reproductive Health in Ethiopia.

[15] UNFPA (2011). People and possibilities in a world of 7 billion, state of world population 2011.

[16] Central Statistical Agency (CSA) [Ethiopia] and ICF International (2012). Ethiopia Demographic and Health Survey 2011.

[17] Central Statistical Agency (CSA) [Ethiopia] and ORC Macro (2006). Ethiopia Demographic and Health Survey 2005.

[18] Tegegn A, Gelaw Y (2009). Adolescet reproductive health services in jimma city: accessibility and utilization. Ethiopian Journal of Health science.

[19] Seifu A, Fantahun M, Worku A (2006). Reproductive health needs of out-of-school adolescents: A cross-sectional comparative study of rural and urban areas in northwest Ethiopia. Ethiopian Journal of Health Development.

[20] Lawrence DE I, Rose T (2007). Sexual initiation and contraceptive use among female adolescents in Kenya. African Journal of Health Sciences.

[21] CorreiaDS, Ana C.P. Pontes, JCC, E. Sócrates T. Egito, Eulália M.C. Maia (2009). Adolescents: Contraceptive Knowledge and Use, a Brazilian Study. ScientificWorldJournal.

[22] Erulkar AS, Mekbib T, Simie N, Gulema T (2006). Differential use of adolescent reproductive health programs in Addis Ababa Ethiopian. J Adolesc Health.

[23] Hall KS, Moreau C, Trussell J (2012). Associations Between Sexual and Reproductive Health Communication and Health Service Use Among US Adolescent Women. Perspectives on sexual and reproductive health.

[24] Ketende C, Gupta N, Bessinger R (2003). Facility-level reproductive health interventions and contraceptive use in Uganda. International family planning perspectives.

[25] Chukwuemeka E. Nwachukwu1, Odimegwu, C (2011). Regional patterns and correlates of HIV voluntary counselling and testing among youths in Nigeria. African journal of reproductive health.

[26] Molla M, Emmelin M, Berhane Y, Lindtjørn B (2009). Perception of Ethiopian youth regarding their risk of HIV: A community-based study among youth in predominately rural Butajira. Ethiopian Journal of Reproductive Health.

[27] Hutchinson PL, Mahlalela X (2006). Utilization of voluntary counseling and testing services in the Eastern Cape, South Africa. AIDS Care.

[28] Biddlecom  AE, Munthali A, Singh S, Woog V (2007). Adolescents’ views of and preferences for sexual and reproductive health services in Burkina Faso, Ghana, Malawi and Uganda. Afr J Reprod Health.

[29] Adebayo O, Clement D, Vincent O, EsiteU, EsiteA, AdebunmiO (2010). Care Seeking Determinants among Adolescents in Lagos, Nigeria. Sierra Leone Journal of Biomedical Research.

